# Enhanced Analgesia and Reduced Opioid Use with Dexmedetomidine-Ropivacaine for Superficial Cervical Plexus Block in Thyroidectomy: A Randomized Controlled Study

**DOI:** 10.1055/s-0046-1819561

**Published:** 2026-05-08

**Authors:** Sucheta Bagri, Rajan B. Godwin, Ashish Gupta, Shivank Sethi, Neeraj Narang, Sanjay Kumar Yadav, Dhananjaya Sharma

**Affiliations:** 1 Department of Anesthesiology, Netaji Subhash Chandra Bose Medical College and Hospital, Jabalpur, Madhya Pradesh, India; 2 Department of Surgery, Netaji Subhash Chandra Bose Medical College and Hospital, Jabalpur, Madhya Pradesh, India

**Keywords:** thyroidectomy, opioid analgesia requirement, bilateral superficial cervical plexus block, dexmedetomidine

## Abstract

**Abstract:**

**Introduction:**

There are concerns about addiction to opioids due to excessive prescription after surgery.

**Objective:**

We compared the impact of dexmedetomidine as an additive to ropivacaine for bilateral cervical plexus block to that of ropivacaine alone for postoperative analgesia in patients undergoing thyroidectomy.

**Methods:**

A total of 60 patients were randomized in 2 groups: those submitted to bilateral superficial cervical plexus block (BSCPB) using 0.375% ropivacaine (group BSCPB-R, the control group) and those submitted to BSCPB using 0.375% ropivacaine with 1mcg/kg of dexmedetomidine (group BSCPB-RD, the study group). The resultant postoperative analgesia was compared by the morphine milligram equivalents (MMEs) required by each patient.

**Results:**

Only 3 (10%) patients in the BSCPB-R group and 2 (6.6%) patients in the BSCPB-RD group required opioid analgesia. The mean total rescue opioid required in the first 24 hours was significantly higher in group BSCPB-R compared to group BSCPB-RD (20 ± 8.14 versus 8 ± 2.85 MME respectively; *p* = 0.0001). Similarly, the mean total MMEs required during the first 5 days after surgery was of 38 ± 16.4 for group BSCPB-R and 18 ± 8 for group BSCPB-RD ( *p* = 0.0001).

**Conclusion:**

The combination of dexmedetomidine and ropivacaine in the BSCPB protocol results in a lower requirement of opioids for pain in the postoperative period, and very few (if any) patients undergoing thyroid surgery require postoperative opioids with this strategy.

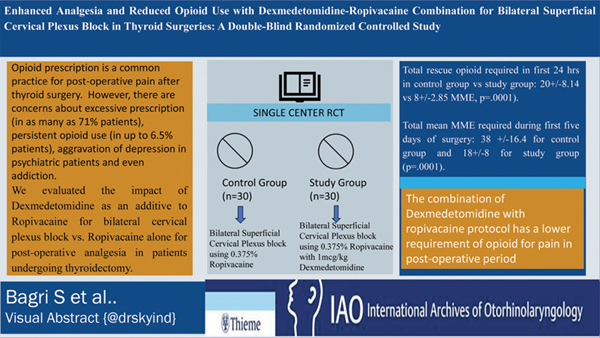

## Introduction

Thyroid surgery is one of the most frequently performed endocrine procedures worldwide. 1 Opioid prescription is a common practice for postoperative pain. However, there are concerns about excessive prescription (in as many as 71% of the patients), persistent opioid use (in up to 6.5%), aggravation of depression in psychiatric patients, and even addiction. 2 3 4 5 This calls for minimum use of opioids and responsible prescription practices on the part of the surgeons. This prompted us to compare the impact of dexmedetomidine as an additive to ropivacaine to that of ropivacaine alone, in terms of postoperative analgesia, for bilateral superficial cervical plexus block (BSCPB) in patients undergoing thyroidectomy.

## Methods

### Design and Participants

The current double-blinded, randomized controlled study was conducted in the Departments of Anesthesiology and Surgery of a tertiary teaching hospital in central India. Approval was obtained from the institutional Ethics Committee of the Netaji Subhash Chandra Bose Medical College, in Jabalpur, India, and informed consent was obtained from all patients.

### Inclusion and Exclusion Criteria

All patients aged between 18 and 65 years, of grades I and II on the American Society of Anesthesiologists (ASA) classification, who were undergoing elective thyroidectomy were included. We excluded patients with a history of neck surgery, surgical-site infection, bleeding disorders, and chronic neck pain, those unable to understand and correctly score their pain, as well as patients who did not provide consent.

### Sample Size, Randomization, Blinding and Technique

The mean dose of opioids prescribed postoperatively is of approximately 200 morphine milligram equivalents (MMEs) in thyroid surgery, but it can be decreased to 100 to 115 MMEs with preoperative patient education and use of nonopioid medications. 4 In total, 60 patients (30 in each group) were required to have a 90% chance of detecting, as significant at the 5% level, a decrease in the primary outcome measure from 200 MMEs in the control group to 112 MMEs in the study group.

All patients underwent a thorough preanesthetic evaluation, and they were explained in detail the anesthetic procedure and the score on the Numeric Rating Scale (NRS), a modified version of the Visual Analogue Scale (VAS) that consists of a segmented numerical scale with 11 points (ranging from 0 to 10), which was used to calculate activity pain. All patients received intravenous midazolam 1mg and intravenous fentanyl 50 μg before performing the block. The patients and the evaluator in the ward were blinded to the group allocation. None of the subjects took analgesics or sedatives other than the premedications administered during the study protocol.

The patients were randomized into 2 groups of 30 each, using a computer-generated randomization table:

- Patients undergoing BSCPB using 0.375% ropivacaine (group BSCPB-R, the control group): 10 mL of 0.75% ropivacaine were diluted to the concentration of 0.375% with 10 mL of distilled water, and 10 mL of this solution were used on each side to achieve BSCPB by the standard technique. 6
- Patients undergoing BSCPB using 0.375% ropivacaine with 1mcg/kg of dexmedetomidine (BSCPB-RD group, the study group): 10 mL of 0.75% ropivacaine were diluted with 9 mL of distilled water and dexmedetomidine 1mcg/kg was diluted with 0.5 mL of distilled water to achieve the final concentration of 0.375% of ropivacaine, and 10 mL of this solution were used on each side to achieve BSCPB by the standard technique. 6

After the BSCPB, all patients underwent general anesthesia with intravenous propofol 2 mg/kg and intubation facilitated with intravenous succinylcholine 1.5 mg/kg. Intraoperative analgesia was provided by the administration of 1 gm of intravenous acetaminophen infusion. Anesthesia was maintained through oxygen and nitrous oxide with isoflurane with minimum alveolar concentrations (MACs) ranging from 0.8 to 1.2. Muscle relaxation was provided with an injection of atracurium 0.5mg/kg. At the end of the procedure, the reversal of muscle relaxation was achieved with injections of neostigmine 2.5 mg and glycopyrrolate 0.5mg. After the successful completion of the surgery, the patients were sent to the recovery room, and they were monitored postoperatively for the first analgesic requirement. For rescue analgesia an infusion of 1 gm of intravenous acetaminophen was tried; if not attained, then, oral tramadol 50 mg. The total opioid requirement in the first 24 postoperative hours recorded: The patients were prescribed up to 3 g of oral acetaminophen per day starting on the first postoperative day. If further analgesia was requested, the subjects were prescribed an oral opioid (tramadol).

### Definitions and Outcomes

The dose of tramadol prescribed during rescue analgesia and the dose consumed during first 5 days were used to calculate the total quantity of opioid consumed. For standardization of data analysis, the total amount prescribed was converted to MMEs by multiplying the total milligrams consumed with a standard conversion factor of 0.2 for tramadol. Any episodes of postoperative nausea and vomiting (PONV) were noted. Pain was assessed through the NRS.

### Statistical Analysis

The categorical variables were reported as proportions and analyzed using the Chi-squared or Fisher's exact tests. The continuous variables were expressed as mean and standard deviation (SD) values. Normally-distributed variables were analyzed using the Student's *t* -test, and non-normally-distributed variables were analyzed using the Mann–Whitney U test. Values of *p* < 0.05 were considered statistically significant. The statistical analysis was performed using the MedCalc online software (MedCalc Software Ltd; https://www.medcalc.org/calc/ ).

## Results

Patient demographics were comparable between the two groups ( Table 1 ). The mean time until the first rescue analgesic requirement was of 18.53 hours in group BSCPB-R and 23.40 hours in group BSCPB-RD ( *p* < 0.0001). Three (10%) patients in the BSCPB-R group and 2 (6.6%) patients in the BSCPB-RD group required opioid analgesia. The total rescue opioid required in the first 24 hours was significantly higher in group BSCPB-R compared to group BSCPB-RD (20 ± 8.14 versus 8 ± 2.85 MME respectively; *p* = 0.0001). The total mean MME required during the first 5 days after surgery was of 38 ± 16.4 for group BSCPB-R and of 18 ± 8 for group BSCPB-RD ( *p* = 0.0001; Table 2 ).

**Table 1:** Demographic and clinical profile of the study sample

	BSCPB-R group (control; n = 30)	BSCPB-RD group (study; n = 30)	*p* -value
**Gender: n (%)**			
Male	15 (50)	14 (47)	0.818
Female	15 (50)	16 (53)	
**Mean age (years)**	39.17 ± 9.04	40.43 ± 8	0.613
**Surgery: n (%)**			
Hemithyroidectomy	13 (43)	13 (43)	1.000
Total thyroidectomy	17 (57)	17 (57)	
**Indication: n (%)**			
Benign	22 (73)	17 (57)	0.197
Malignancy	8 (27)	13 (43)	
**Mean operative time (minutes)**	120.83 ± 36	113.67 ± 31.8	0.417
**Mean time until the first rescue analgesic requirement (minutes)**	142 ± 18	136 ± 22	0.252

**Abbreviations:** BSCPB-R, bilateral superficial cervical plexus block with ropivacaine; BSCPB-RD, bilateral superficial cervical plexus block with ropivacaine and dexmedetomidine.

**Table 2:** Mean analgesia requirements in the control and study groups

Variable	BSCPB-R group (control)		BSCPB-RD group (study)		*p* -value
			
Mean total analgesia requirement in the first 24 hours (tramadol, in MME)	20 ± 8.14		8 ± 2.85		*p* < 0.0001; 95%CI: −15.1519 to −8.8481
Mean total opioid quantity required during the next 4 days after surgery (MME)	98 ± 20.3		80 ± 16.8		*p* = 0.0004; 95%CI: −27.6300 to −8.3700
Mean total opioid quantity (MME)	118 ± 26.4		88 ± 18.0		*p* = 0.0001; 95%CI: −41.6774 to −18.3226

**Abbreviations:** BSCPB-R, bilateral superficial cervical plexus block with ropivacaine; BSCPB-RD, bilateral superficial cervical plexus block with ropivacaine and dexmedetomidine; MME, morphine milligram equivalents.

**Fig. 1:**
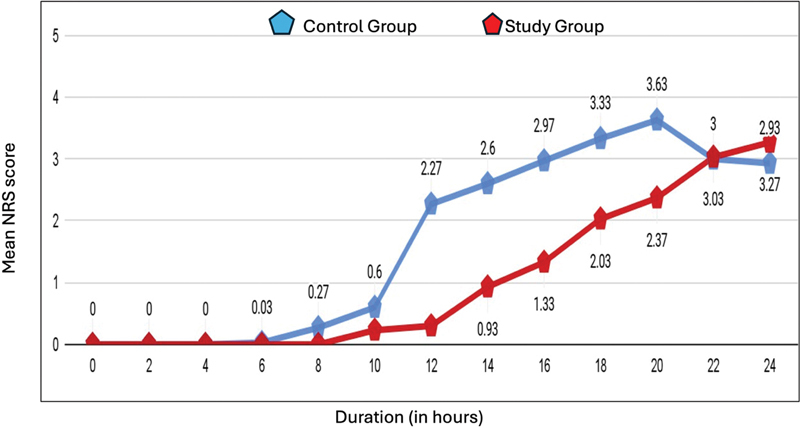
Postoperative scores on the Numeric Rating Scale (NRS) pf the control and study groups.

**Fig. 2:**
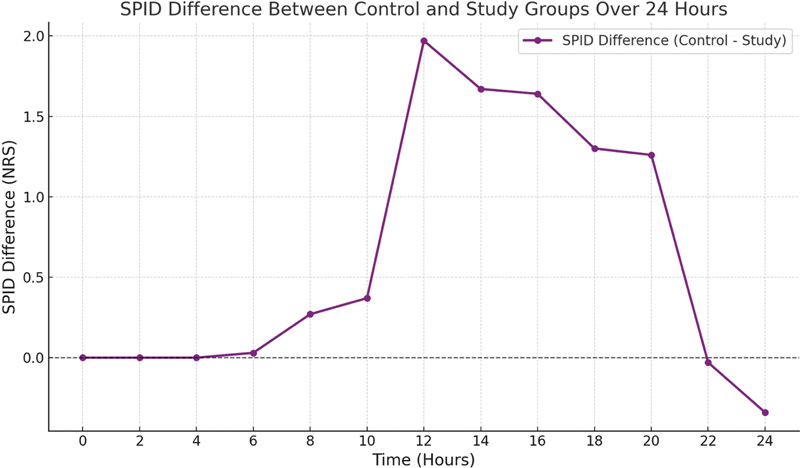
Graphical representation of the Sum of Pain Intensity Difference (SPID) between the control and study groups throughout 24 hours.

The NRS scores were significantly lower in group BSCPB-RD during the first 20 hours ( Table 3 ; Fig. 1 ). Fig. 2 displays the Sum of Pain Intensity Difference (SPID) between the control and study groups throughout 24 hours: the study group experienced significantly better pain relief than the control Group, especially during the intermediate postoperative period (6 to 20 hours). Overall, 5 (16.5%) patients in each group experienced postoperative nausea and vomiting ( *p* = 1).

**Table 3:** Mean postoperative NRS scores of the control and study groups

Mean NRS score	N	BSCPB-R group (control)		BSCPB-RD group (study)		*p* -value
			
At 0 hour	30	0.00 ± 0.00		0.00 ± 0.00		
After 2 hours	30	0.00 ± 0.00		0.00 ± 0.00		
After 4 hours	30	0.00 ± 0.00		0.00 ± 0.00		
After 6 hours	30	0.03 ± 0.18		0.00 ± 0.00		0.321
After 8 hours	30	0.27 ± 0.45		0.00 ± 0.00		0.002
After 10 hours	30	0.60 ± 0.50		0.23 ± 0.43		0.003
After 12 hours	30	2.27 ± 0.58		0.300.47		< 0.0001
After 14 hours	30	2.60 ± 0.62		0.93 ± 0.58		< 0.0001
After 16 hours	30	2.97 ± 0.72		1.33 ± 0.71		< 0.0001
After 18 hours	30	3.33 ± 0.48		2.03 ± 0.61		< 0.0001
After 20 hours	30	3.63 ± 0.49		2.37 ± 0.76		< 0.0001
After 22 hours	30	3.00 ± 0.87		3.03 ± 0.56		0.86
After 24 hours	30	2.93 ± 0.64		3.27 ± 0.52		0.031

**Abbreviations:** BSCPB-R, bilateral superficial cervical plexus block with ropivacaine; BSCPB-RD, bilateral superficial cervical plexus block with ropivacaine and dexmedetomidine; NRS, Numerical Rating Scale.

## Discussion

The present double-blinded, randomized controlled study has shown that the addition of dexmedetomidine to ropivacaine for BSCPB in thyroid surgeries significantly decreased the opioid consumption and is associated with better analgesia during the first 24 hours after surgery.

The enhanced analgesic effect observed with the combination of dexmedetomidine and ropivacaine is likely due to the synergistic actions of the two drugs. Dexmedetomidine, an alpha-2 adrenergic agonist, provides sedation, anxiolysis, and analgesia without significant respiratory depression, while ropivacaine, a long-acting local anesthetic, offers prolonged nerve blockade. The combination offers superior pain control compared to ropivacaine alone, aligning with the growing interest in multimodal-analgesia strategies to minimize opioid use and its associated risks. 3 4 Our findings are consistent with those of previous studies 6 7 that have demonstrated the benefits of using dexmedetomidine as an adjuvant in regional anesthesia.

The BSCPB has become an accepted intervention which significantly reduces pain intensity and opioid requirement after thyroid surgery. 8 9 10 11 Debates about the usefulness of bupivacaine wound infiltration 12 and pre- or postoperative BSCPB 13 have been settled by a recent meta-analysis 14 that has shown that preoperative interventions have more advantages than local wound infiltration and postthyroidectomy intervention in alleviating patients' pain.

Patients undergoing thyroid or parathyroid surgery are at high risk of developing postoperative nausea and vomiting. 15 Hence any intervention that reduces the opioid requirement is highly desirable. Overall, most (93%) patients undergoing thyroidectomy and parathyroidectomy present very small opioid requirements; hence, these patients can be discharged with doses of opioids ≤ 20 MME. 16 This leads to decreased opioid requirement and increased patient safety. Implementation of an enhanced recovery after surgery (ERAS) protocol can also decrease opioid requirements. 17 In the current study, the total MME (inpatient plus discharge) was brought down to 12 in the first 24 hours and to 20 five days after surgery.

These results should be viewed with some limitations. First, pain between both groups was only compared for the first 24 postoperative hours, but not after that. Second, the sample size of 60 subjects is comparatively small and may limit the generalizability of ours findings. Another limitation is that due to logistic reasons (discharge of the patients), we could not calculate opioid consumption beyond 5 days. However, the strengths of the current study include its randomized controlled design, which enhances the validity of our conclusions. This study's innovation lies in its focus on thyroidectomy, a surgical procedure with unique analgesic challenges. It is crucial to control postoperative pain to prevent increased morbidity, delayed recovery, a delayed return to normal daily living, a reduction in patient satisfaction, and increases in the use of health care resources and health care costs. 18 Future studies should aim to replicate our findings in larger, more diverse patient populations and investigate the optimal dosing strategies and potential side effects of this combination to further refine its clinical use.

## Conclusion

The combination of dexmedetomidine and ropivacaine for the BSCPB protocol results in a lower requirement of opioids for postoperative pain, and very few (if any) patients undergoing thyroid surgery require postoperative opioids with this strategy.
